# Study of Gases Permeation in Necklace-Shaped Dimethylsiloxane Polymers Bearing POSS Cages

**DOI:** 10.3390/membranes9040054

**Published:** 2019-04-16

**Authors:** Roman Selyanchyn, Shigenori Fujikawa, Naohiro Katsuta, Kazuya Suwa, Masashi Kunitake

**Affiliations:** 1 WPI International Institute for Carbon-Neutral Energy Research (WPI-I2CNER) Kyushu University, 744 Motooka, Nishi-ku, Fukuoka 819-0395, Japan; fujikawa.shigenori.137@m.kyushu-u.ac.jp; 2 NanoMembrane Technologies Inc., 4-1, Kyudai-Shimachi, Nishi-ku, Fukuoka 819-0388, Japan; 3 Center for Molecular Systems (CMS), Kyushu University, 744 Motooka, Nishi-ku, Fukuoka 819-0395, Japan; 4 Laboratory for Chemistry and Life Science, Tokyo Institute of Technology, 4259 Nagatsutacho, Midori-ku, Yokohama 226-8503, Japan; 5 Faculty of Advanced Science and Technology, Kumamoto University, 2-39-1 Kurokami, Chuo-ku, Kumamoto 860-8555, Japan; 159d8309@st.kumamoto-u.ac.jp; 6 JNC Petrochemical Corporation, Ichihara Research Center; 5-1 Goikagan, Ichihara, Chiba 290-8551, Japan; k.suwa@jnc-corp.co.jp

**Keywords:** POSS, organic–inorganic hybrid, necklace shaped polymer, gas separation membrane

## Abstract

The transport of small gases (H_2_, CO_2_, N_2_, O_2_) through a series of novel membranes based on necklace-shaped inorganic polymers (DMS@POSS), in which a polyhedral oligomeric silsesquioxane (POSS) cage unit and soft chains of oligo-dimethyl siloxane (DMS) were alternately connected, was investigated. The influence of the DMS chain length and crosslinking density of the DMS@POSS on membrane properties were studied. The membranes revealed characteristic structure-property relation towards both glass transition and gases transport. Specifically, clear dependence of properties from the length of DMS units (or overall siloxane content) was revealed. Gas transport properties, when compared to state-of-art polydimethylsiloxane and commercial silicone rubber, demonstrated significantly higher selectivity of DMS@POSS for carbon dioxide (in CO_2_/N_2_), hydrogen (in H_2_/N_2_) and oxygen (in O_2_/N_2_) but lowered permeability, proportional to the amount of POSS in the material. With a precise control over mechanical and thermal properties compared to conventional silicone rubbers, described materials could be considered as materials of choice in niche gas separation or other applications.

## 1. Introduction

Among the huge structural diversity of siloxane derivatives, polyhedral oligomeric silsesquioxane (POSS) molecules, which possess discrete nanocage structures, have been attracting significant fundamental and industrial interest [1]. Distinctive features of the POSS molecules (particles) are their size in the range of 1–3 nm and the possibility of tailoring their properties by the introduction of various functional groups on the surface. These feature make them very attractive candidates for fabrication of organic–inorganic hybrid nanocomposites [2]. For instance, POSS molecules, when used as fillers or components in polymer matrices, can provide gains in thermal stability [3] or modify dielectric properties [4], generally without a big influence on transparency [5]. 

Recently, the necklace-shaped polymers, in which a “hard and bulky” unit and a “soft” chain such as POSS and dimethyloligosiloxane (DMS) respectively, are alternately bonded in the polymer main chain, have attracted much attention as a primary polymer design leading to well-balanced nano-fusion. We have reported the alternating siloxane copolymers consisted of a bifunctional POSS cage and a flexible linear DMS chain segment [6,7,8]. The necklace-shaped linear polymers (thermoplastics), DMS@POSS, are synthesized via stepwise polycondensation or ring opening (equilibrium) polymerization with a bifunctional POSS cage molecule as a key precursor. Two synthetic routes allowed us to produce the series of the polymers with structural varieties in terms of chain arrangements, namely the chain length between POSS units, type of arrangement (fixed modulated and random), and the average molecular weight. The library of polymers with structural variation are optimal to explore the correlation between the primary nanostructure and properties of the polymers. For instance, the correlations between the average length of the DMS chains and thermal properties such as glass transition temperatures and decomposition temperature have elucidated the structures with significantly high heat resistance [8]. Other properties of POSS nanocomposites are yet to be investigated. In particular, the transport of gases through the POSS containing hybrids is an important direction of research because these materials could provide necessary alternatives over conventional organic polymers. 

Several studies on gas permeation using polymer membranes containing POSS units have been reported. For instance, Rahman et al. prepared nanocomposite membranes by incorporation of poly(ethylene glycol) functionalized POSS (PEG-POSS) in commercial poly(ether-block-amide) (Pebax^®^ MH 1657). They demonstrated a two-fold increase in CO_2_ permeability without significant influence on selectivity while other composite (with Pebax^®^ 2533) showed enhancement in both selectivity and permeability upon up to 30 wt% POSS incorporation [9]. Raaijmakers et al. synthesized hyper-cross-linked polyPOSS−imide membranes with tailored intercage spacing showing that length and flexibility of their imide bridges enabled tuning of gas permeability and selectivity in a broad temperature range [10,11]. 

As outlined by Madhavan et al. [2], in regard to gas separation, POSS derivatives have a unique feature being an intermediate between that of silica (SiO_2_) and siloxane (R_2_SiO). Due to utilization of DMS connection between POSS particles, the necklace-shaped DMS@POSS polymer materials that we developed are even closer to the family of the polydimethylsiloxanes. Therefore, knowledge of gas permeation in these hybrids is of high importance. Precise understanding of the relationship of a structure and gas permeability property can show the way towards mechanically and thermally stable membrane materials. Few studies have reported the composite materials that are based on combination of POSS and PDMS. Chen et al. [12] used divinyl-hexa[(trimethoxysilyl)ethyl]-POSS as an alternative cross-linker of the PDMS and demonstrated the improvement of the thermal stability and mechanical properties (tensile strength, modulus, and hardness) with the increase of modified POSS loading up to 20%. In other works, researchers have blended heptaphenylhydrogen silsesquioxanes with high molecular weight PDMS and also observed the gradual change of thermal properties and crystallization behavior with the loadings up to 10 wt% [13]. Several studies have also investigated the gas permeability in the composites based specifically on POSS and PDMS. Madhavan et al. [2] reported the decrease of permeability in composites containing CyPOSS (partially caged heptacyclopentyl tricycloheptasiloxane triol) in PDMS-polyurethane (PDMS-PU) matrix. With the increase of loading from 5% to 25%, permeability of CO_2_, O_2_ and N_2_ decreased, while CO_2_/N_2_ and O_2_/N_2_ selectivities demonstrated an increase in selectivity. Interestingly, when the amine functionalized POSS was introduced in similar PDMS-PU matrix both permeability and selectivity decreased with the content of POSS-amine from 5% to 25% [14]. The decrease of H_2_ and CO_2_ permeabilities was observed in the work of Rezakazemi [15] who used 2–6% of octatrimetoxy-POSS as filler. 

**Figure 1:**
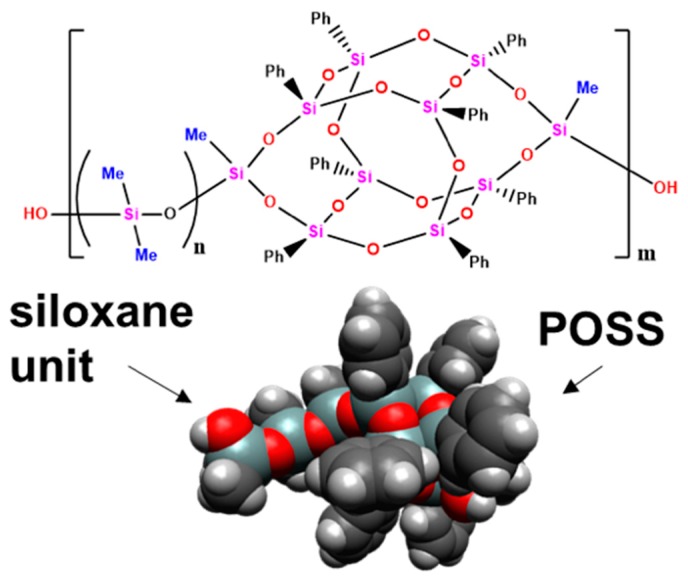
Chemical structure and 3D representation of the repeating unit in the DMS@POSS polymers.

In this work, the transport of small gases through the series of cross-linked polymer membranes based on necklace-shaped DMS@POSS polymers (chemical structure given in Figure 1) was studied. The thermosetting network polymers were prepared by crosslinking the silanol groups at the both ends of the DMS@POSS macromers. By crosslinking only at both the ends of the polymers, it is possible to retain the basic physicochemical properties of the original necklace-shaped polymers even in the polymer networks. Variation of the DMS length and amount of crosslinker were used as parameters for material variation.

## 2. Materials and Methods

### 2.1. Membranes Fabrication

The synthesis and characterization of bifunctional POSS molecules and necklace-shaped POSS-DMS polymers with “constant chain” and “random chain” arrangements by polycondensation and ring opening polymerization have been reported in earlier works [6,8]. The membranes, ${\mathrm{DMS}}_{y}^{x}@\mathrm{POSS}$ (where x is the average DMS length and y is the crosslinking ratio) were prepared by casting of THF solution of a polymer precursor with a multi-functional silane cross-linking agent MS-51 (Mitsubishi Chemical Corp., Tokyo, Japan) and dibutyltin dilaurate (Catalyst, Tokyo Chemical Industry Co., Ltd., Tokyo, Japan) into a Teflon dish followed by heat treatment at 200 °C. The catalyst concentration was typically 0.021 ppm by weight relative to the macromer. All of the polymer membranes used were transparent, indicating amorphous structures. After crosslinking completion materials are stable in normal environment and are not affected by environmental factors (e.g., high humidity or high temperature). Table 1 provides the summary of the materials used in this study.

### 2.2. Characterization

The chemical structure of fabricated composite membranes was analyzed by Fourier transform infrared spectroscopy (FTIR) using a scanning FTIR Microscope (Nicolet iN10 MX, ThermoFischer, Tokyo, Japan). The test was conducted in the attenuated total reflectance (ATR) mode using actual membranes for analysis in the scanning range of 4000–650 cm^−1^ with a resolution of 4 cm^−1^. Pressure was changed accordingly with samples’ hardness in order to obtain maximum contact to obtain reliable spectra. 

Differential scanning calorimetry (DSC) was carried out using a NETZSCH instrument (DSC 204 F1 Phoenix^®^, NETZSCH, Selb, Germany) under a nitrogen atmosphere (50 mL·min^−1^). The samples in an aluminum pan (mass ~3 mg) were cooled below the glass transition point (T_g_) and re-heated to well above T_g_. The heating and cooling rate was set to 30 °C·min^−1^ with an empty aluminum pan used as a reference. The T_g_ value was determined as the midpoint value between the onset and the end of a step transition using the NETZSCH analysis software (Proteus Analysis, version 7.1.0).

### 2.3. Gas Permeability Measurement

**Figure 2:**
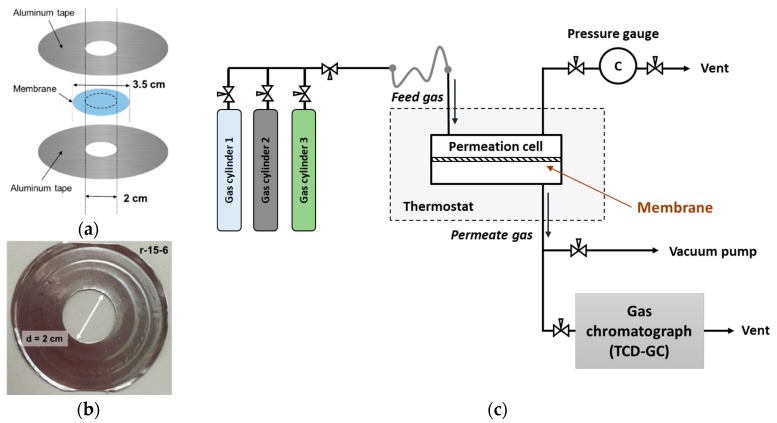
(**a**) Schematic image of DMS@POSS membrane assembly for the gas transport measurement in GTR-TEC machine; (**b**) Photography of the typical sample used for gas permeability measurements (**c**) schematic image of the steel cell used for gas transport measurement in the GTR-TEC device.

For the gas permeation measurements, the polymer membranes were masked with alumina tape (Bytac^®^) to provide an open circle of 2 cm diameter (a respective active surface area ~ 3.14 cm^2^) as schematically shown in Figure 2a and optical photography of the actual sample in Figure 2b. Dry gases permeation rates were measured at room temperature using GTR-11A/31A gas barrier testing system (GTR Tec Corp., Kyoto, Japan) schematically given in Figure 2c. This system has three independent test cells and operates the differential pressure method to measure the gas permeation rates (compliant with ISO 15105-1 and JIS K7126 standards). In the machine, gas transport through the membrane is induced by initial vacuum applied on the permeate side and gas compression applied on the feed side once the valves are open. For all measurements, the standard total pressure difference was set to 200 kPa. Gas collected in the test cell is automatically transferred to a gas chromatograph equipped with thermal conductivity detector (TCD, G3700T, Yanaco, Kyoto, Japan) where total volume is detected respectively to previously measured calibration line. Sample collection time was adjusted along with the permeation properties of the membrane in a way to avoid significant change of transmembrane pressure.

Thickness of the membranes was measured by digital micrometer (Mitutoyo, Japan) and was in the range 120–160 µm. A commercial silicone rubber membrane (Togawa Rubber Ltd., Japan) with thickness of 500 µm was used as a reference. Gas permeation was measured for pure gases purchased from (Fukuoka Sanso, Fukuoka, Japan) and each membrane sample was measured in several replicates (5–15). Average values of permeability (*p*) in barrer units (1 barrer = 10^−10^ cm^3^(STP)·cm/cm^2^·s·cmHg) was calculated and is reported. The ideal selectivity between two different gases in a composite membrane was calculated by taking the ratio of the permeabilities of different gases, *α*_ij_ = *p*_i_/*p*_j_, where *p*_i_ and *p*_j_ are the permeabilities of fast gas *i* and slow gas *j*, respectively.

## 3. Results

### 3.1. Membranes Characterization

**Figure 3:**
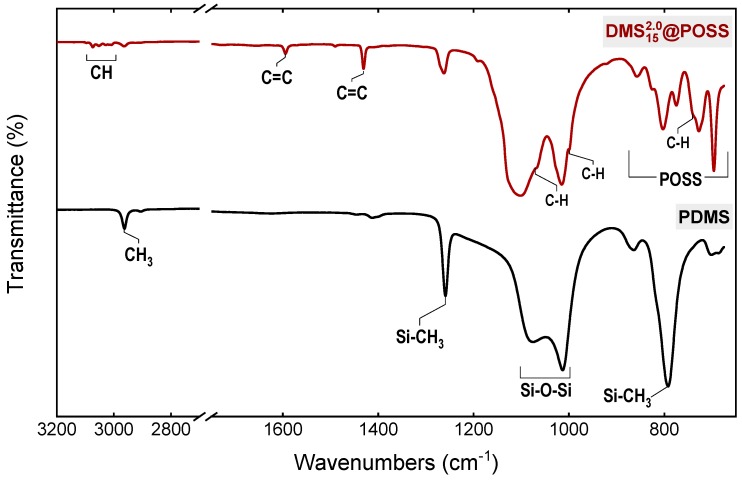
ATR-FT-IR spectrum of the ${\mathrm{DMS}}_{15}^{2.0}@\mathrm{POSS}$ membrane (upper spectrum) compared to a spectrum of conventional silicone rubber membrane (bottom spectrum).

Figure 3 shows the typical ATR-FTIR spectra obtained for the ${\mathrm{DMS}}_{y}^{x}@\mathrm{POSS}$ and PDMS (silicone) membranes. As seen, due to presence of methyl siloxane linkers between phenyl-POSS particles, ${\mathrm{DMS}}_{y}^{x}@\mathrm{POSS}$ sample includes all signals characteristic for the polysiloxane, as indicated in the bottom plot in Figure 3, particularly intense asymmetric Si-CH_3_ stretching (around 2960 cm^−1^), symmetric CH_3_ deformations (1258 cm^−1^) Si-C stretching (790 cm^−1^), Si-O-Si stretching (1010 and 1075 cm^−1^). Additionally, the ${\mathrm{DMS}}_{y}^{x}@\mathrm{POSS}$ samples clearly show the signals originating from the incorporated phenyl-POSS, such as CH stretching in aromatic ring (3000–3100 cm^−1^), C-C stretching in the aromatic ring (1600 cm^−1^ and 1430 cm^−1^), in-plane CH bending (shoulders around 1000 and 1070 cm^−1^), out of plane CH bending (shoulder around 740 cm^−1^), as well as more complex Si-O-Si signals in the range of 650–900 cm^−1^ characteristic to POSS cages. Qualitatively, all samples used in the work give similar FTIR spectral results, proving that, structurally, materials are similar to each other. Other spectra are therefore not shown for clarity in this work.

**Figure 4:**
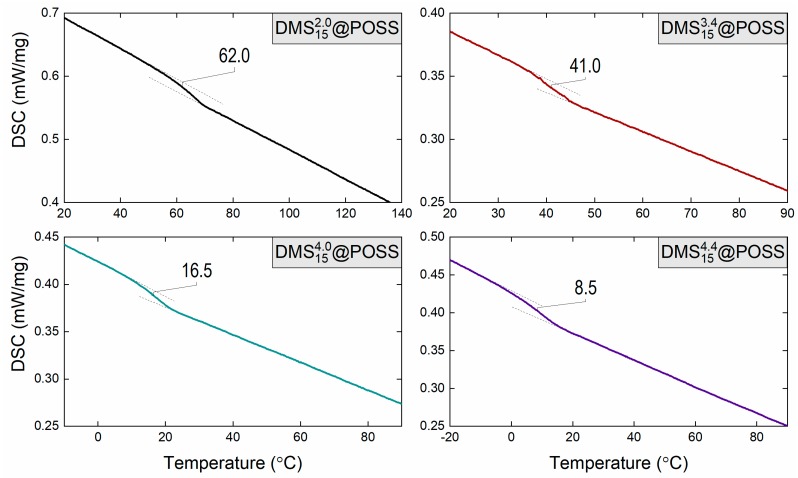
Dynamic scanning calorimetry results obtained for the ${\mathrm{DMS}}_{y}^{x}@\mathrm{POSS}$ composite membranes demonstrating clear glass transition behavior (T_g_ indicated on each plot) which correlates with the material composition, namely higher T_g_ corresponds to higher amount of POSS in material.

In our earlier work, glass transition temperatures (T_g_) were measured by thermal mechanical analysis (TMA), in which T_g_ is detected using the indentation probe during the material gradual heating [8]. Here, we have conducted additionally dynamic scanning calorimetry to confirm the T_g_ of the actual membrane samples. The result, given in Figure 4, shows clear glass transitions taking place in all samples. As seen T_g_ value is proportional to the amount of POSS in materials (inversely proportional to the length of DMS linker.) This structure-property relation provides an important demonstration of the control over thermal properties one obtains by changing the way how material is synthesized.

The glass transition temperatures determined using TMA and DSC were in good agreement except for ${\mathrm{DMS}}_{15}^{2.0}@\mathrm{POSS}$ bearing the shortest DMS chain length (summarized in Table 2). DSC and TMA measurements are methodologies to observe the melting of polymer chains and the softening of the materials from microscopic and macroscopic aspects, respectively. In previous research, it was found that the POSS-DMS alternating polymer of fixed chain length of 2 can reveal crystallinity, although other POSS polymers bearing longer DMS chains demonstrate amorphous nature. This relatively low dispersibility of the POSS units in the polymer matrix may be considered the reason for the difference in apparent T_g_.

### 3.2. Gases Permeation at Room Temperature

**Figure 5:**
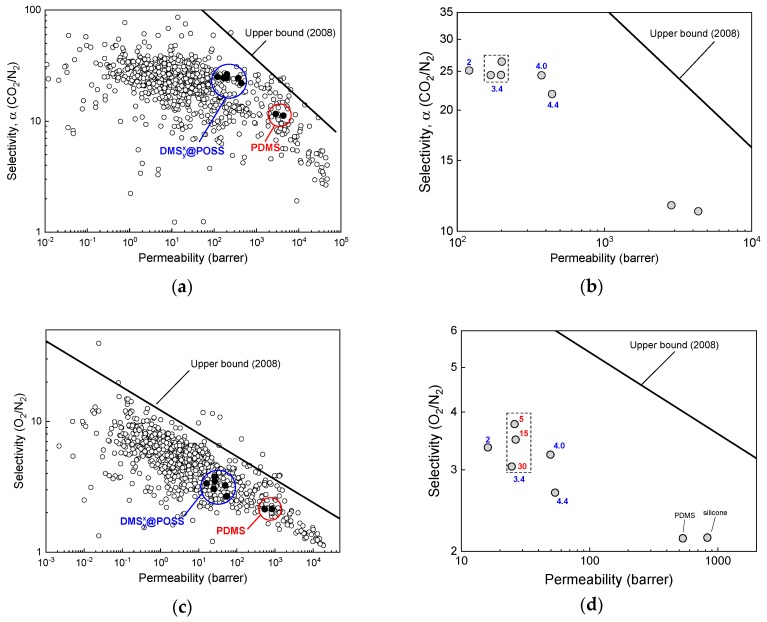

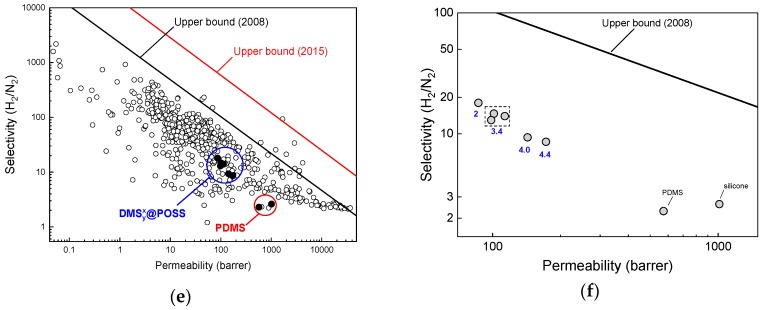
(**a**) Carbon dioxide/nitrogen selectivity as a function of carbon dioxide permeability for ${\mathrm{DMS}}_{y}^{x}@\mathrm{POSS}$ samples (blue circle outline) polydimethylsiloxane and commercial silicone (red circle outline) compared to the wide variety of organic polymers published in literature [16] and Robeson upper bound [17]; (**b**) Same data represented in smaller scale to visualize the difference between different materials (blue number next to the data point indicated the lengths of the DMS link, *x* in the material); (**c**,**d**) similar results for oxygen/nitrogen pair (red numbers indicate crosslinking density, *y*); (**e**,**f**) similar results for hydrogen/nitrogen pair.

Figure 5 summarizes the measured pure gases selectivity as a function of permeability of fast gas for the case of the most important from the industrial point of view, namely CO_2_/N_2_ (post combustion CO_2_ capture at heat power plants), O_2_/N_2_ (oxygen enrichment/ nitrogen purification), and H_2_/N_2_ (hydrogen purification) as gas pairs for separation. Table 3 provides numerical values of the same data. In Figure 5a,c,e the data measured for ${\mathrm{DMS}}_{y}^{x}@\mathrm{POSS}$ membranes is compared with the database of measured organic polymer membranes reported in the literature so far [16], together with the most recent Robeson upper bound established for the organic polymer membranes [17].

A detailed result for the CO_2_/N_2_ separation by ${\mathrm{DMS}}_{y}^{x}@\mathrm{POSS}$ membranes shows significantly higher selectivity to CO_2_ for all membranes (α~25) compared to conventional PDMS [18] and commercial silicone (α~11). However, at the same time, noteworthy reductions of permeability were observed for all membranes, namely from ca. 440 barrers (in ${\mathrm{DMS}}_{15}^{4.4}@\mathrm{POSS}$) to ca. 120 barrers (in ${\mathrm{DMS}}_{15}^{2.0}@\mathrm{POSS}$). Such behavior may be related to the increased content of the POSS component and/or decreased DMS content in the different samples. Polydimethylsiloxane is known as highly permeable material [19]; therefore, it is plausible to relate the increased permeability with the increase of DMS content in the membrane material. 

One more observation that is given in Figure 5b shows no significant differences between the ${\mathrm{DMS}}_{y}^{3.4}@\mathrm{POSS}$ membrane samples in respect to the cross-linking ratio. These results indicate that sufficient three-dimensional polymer networks were constructed even with a relatively low degree of crosslinking. This is a promising finding as the crosslinking amount that is usually important for control of mechanical properties of membranes may also substantially reduce the permeability [20]. Overall, for the CO_2_/N_2_ pair separation, we can say that ${\mathrm{DMS}}_{y}^{x}@\mathrm{POSS}$ membranes perform better than the majority of conventional polymers, and have suitable separation parameters.

For the case of O_2_/N_2_ pair separation, all tested ${\mathrm{DMS}}_{y}^{x}@\mathrm{POSS}$ membranes, similar to previous gas pair, demonstrated decreased permeability/increased selectivity compared to conventional PDMS samples. However, contrary to the CO_2_/N_2_ data we do see some influence of crosslinking on the gas permeation for ${\mathrm{DMS}}_{y}^{3.4}@\mathrm{POSS}$ membranes. Namely, denser crosslinking leads to the decrease in both selectivity and permeability which is expected behavior as the crosslinking ratio may reduce the free volume of the polymer reducing the diffusivity of gases. This finding also suggests the different pathway of gas permeation compared to CO_2_ which didn’t demonstrate such behavior.

Finally, for the H_2_/N_2_ separation given in Figure 5e,f, we observe clear trade-off behavior, namely samples with the highest selectivity show the smallest permeability and vice versa. The increase in selectivity for hydrogen over nitrogen compared to conventional materials (5–10 times) is highest among three gas pairs of industrial interest. This means that the structural difference of the membranes is less notable for hydrogen gas where permeability decreased 4–8 times compared to PDMS. However, it is unlikely that H_2_ can penetrate the POSS cages as the smallest gas because in such case we should have observed much better permeability expected for materials with fine micropores [21].

**Figure 6:**
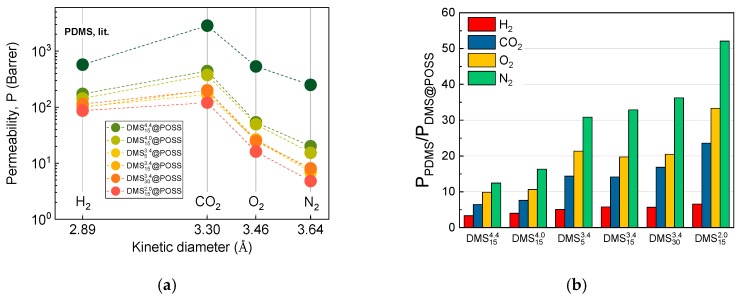
(**a**) Permeability of measured gases (H_2_, CO_2_, O_2_, N_2_) in ${\mathrm{DMS}}_{y}^{x}@\mathrm{POSS}$ membranes as a function of gas kinetic diameter compared to the PDMS [18], demonstrating similar pattern, however with a much higher permeability decrease compared to PDMS for larger gases evidencing influence of molecular size on permeability change; (**b**) Factor showing permeability decrease in all membranes compared to PDMS (for all gases).

In order to clarify the dependence of gases permeability in the fabricated ${\mathrm{DMS}}_{y}^{x}@\mathrm{POSS}$ membranes two more dependencies are given, in Figure 6—permeability as a function of gas kinetic diameter and Figure 5—permeability as a function of DMS linker length (mass content of DMS in the material). As seen in Figure 6a, permeability for different gases in ${\mathrm{DMS}}_{y}^{x}@\mathrm{POSS}$ membranes follows a similar pattern as in conventional PDMS, i.e., the most permeable gas is carbon dioxide and the least permeable is nitrogen. However, if we co-relate the changes in the ${\mathrm{DMS}}_{y}^{x}@\mathrm{POSS}$ membranes with PDMS we can see a clear trend shown in Figure 6b, namely the highest change (decrease) of permeability takes place in the membranes where POSS particles are linked with the shortest possible DMS connection (${\mathrm{DMS}}_{15}^{2.0}@\mathrm{POSS}$). Moreover, we observe the correlation of the change with gas kinetic diameter, i.e., the larger is the gas molecule, the bigger is the change (decrease) of permeability relative to PDMS alone. This reflects more clearly the suggested above that the presence of phenyl terminated POSS particles in the membrane is having the smallest influence on the permeation of the smallest gas molecule (H_2_), and largest for the largest (N_2_); that is why the separation of H_2_ and N_2_ is most improved for this pair compared to ordinary PDMS, as shown in Figure 5f.

**Figure 7:**
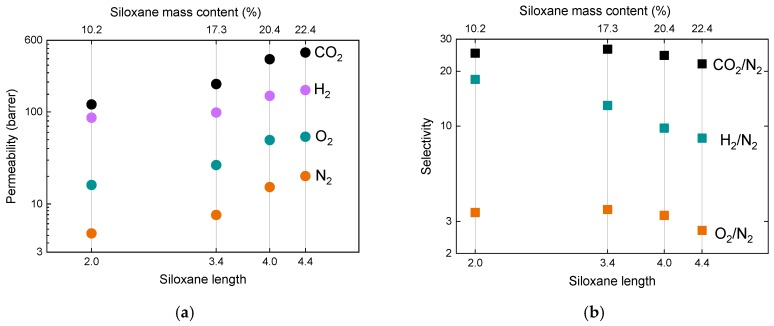
(**a**) Permeability of measured gases (H_2_, CO_2_, O_2_, N_2_) in DMS@POSS membranes as a function of DMS linker length (or total DMS mass content, upper x-scale) suggesting that it is the main factor defining the gas transport through the membranes; (**b**) Selectivity of the important gas pairs as a function of DMS content.

Finally, permeability and selectivity dependences on siloxane linker length (DMS mass content) were built and are shown in Figure 7. Here, we can see a much clearer correlation of the permeability change with the length of DMS linker or mass content of DMS (mass content of POSS) given in Figure 5a. This confirms the idea that the higher the amount of POSS present in the membrane, the slower the gas transport which plausibly happens mainly through the siloxane component of hybrid membranes. A similar conclusion can be given for the selectivity dependence (Figure 7b), namely that it increases with the mass content of POSS for each relevant gas pair.

### 3.3. Influence of the Temperature on the Gas Transport in $DMS_{y}^{3.4}@POSS$ Membranes

**Figure 8:**
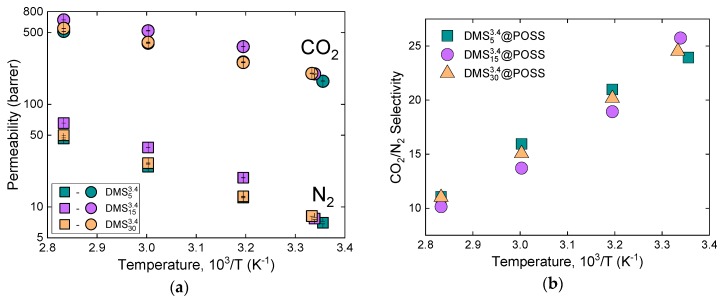
(**a**) Arrhenius plot for three ${\mathrm{DMS}}_{y}^{3.4}@\mathrm{POSS}$ membranes with different crosslinking ratio (permeability dependence on temperature); (**b**) selectivity dependence on temperature.

A unique feature of the used DMS@POSS materials is their glass transition temperatures as given in Figure 4 and Table 2, lies close to the room temperature [8]. CO_2_ and N_2_ permeability of three ${\mathrm{DMS}}_{y}^{3.4}@\mathrm{POSS}$ membranes were investigated in the broader temperature range, covering both glassy (T < T_g_) and rubbery conditions (T > T_g_) of the polymers. Results of both gases and selectivity dependence on temperature are given in Figure 8. Here, we see the behavior expected for the majority of organic polymers i.e., increase of permeability and decrease of selectivity when the temperature is increased. This result confirms that the gas transport in these membranes is mainly governed by the DMS moieties. However, due to confinement of DMS between less permeable (or non-permeable) POSS particles activation energies of permeation (E_P_) are much higher for both nitrogen and carbon dioxide compared to PDMS [18,22] (details in Table 4).

## 4. Discussion

Few studies that have investigated the gas permeability in the POSS-containing composite materials can be found in the literature. To the best of our knowledge, our study is the first report of the gas permeability of the hybrids containing higher amount of POSS compared to amount of polymeric linkers. In the ultimate case, our ${\mathrm{DMS}}_{15}^{2.0}@\mathrm{POSS}$ hybrid membrane contains 88.3 wt% of POSS, while reference studies using the composites where polymer matrix contains smaller amount of POSS. For instance, Madhavan et al. used 5–25 wt% of CyPOSS (partially caged heptacyclopentyl tricycloheptasiloxane triol) and 2.5–7.5 wt% of POSS-H (fully caged octakis(hydridodimethylsiloxy) octasilsesquioxane) fillers in PDMS-polyurethane (PU) matrix [2]. In the work of Guerrero et al. [23], the loading of amidine and lactamide functionalized POSS in polyvinyl alcohol (PVA) matrix was changed in the range from 5 wt% to 50 wt%. Similarly, Rahman et al. used maximum of 30% loading of PEG modified POSS in the CO_2_ philic polymers (Pebax^®^ MH 1657 and Pebax^®^ 2533) [9]. 

Regarding the gas permeability in the POSS-containing materials, membranes comprising CyPOSS in PDMS-PU demonstrated a similar trend as observed in our study, i.e., significant decrease of the CO_2_ permeability with the increased loading of the POSS increasing in the range from 5 to 25 wt% [2]. The same trend was reported in case of amidino-POSS and lactamide-POSS with the increase of loading [20] as well as for other PDMS based composites with relatively smaller amount of POSS [14,15]. Unfortunately, none of these studies investigated the permeation of gases with different kinetic diameters. Increase of the permeability was observed only in case of PEG-modified POSS incorporated in Pebax polymers, however there was no selectivity increase depending on the POSS loading [9] suggesting that separation is still governed solely by the Pebax polymer matrix which is a well-known CO_2_ philic polymer [24]. In our work, both gas permeability and selectivity depend on the amount of siloxane (or the amount of POSS) in the material. However, the dependence of the permeability on loading suggests that transport still takes place only through the siloxane part. Also, in contrast to examples from the literature, we observed improvement of the selectivity which is proportional to the amount of POSS component and highest improvement is observed for the hydrogen/nitrogen pair. As one could notice, selectivity improvement is proportional to the differences of kinetic diameter between two molecules under separation. 

Based on the presented experimental data, the transport of the gas molecules in current DMS@POSS composites takes place in the confined space filled with siloxane. Confined space adds significantly to the size sieving phenomena, allowing faster diffusion of small molecules. As a result, in the materials presented, two factors are defining the properties: size sieving around the densely packed POSS particles and solution-diffusion through the siloxane that links POSS particles together. Therefore, we have to conclude that the mass (volume) content of the siloxane in the material was the most significant factor for the gas permeation. Similarly, in the work of Guerrero et al. CO_2_ permeability change was only correlated to the degree of crystallinity of the POSS containing composite and no influence of the molecular structure and the type of modification was observed [23]. 

## 5. Conclusions

Investigation of gas transport in the novel ${\mathrm{DMS}}_{y}^{x}@\mathrm{POSS}$ membranes showed the decrease of permeability for all gases that was proportional to the increase of POSS loading. Similarly to other POSS based membranes reported in the literature [10,11,21], it looks that the POSS components including the diagonally protruded phenyl units are not accessible to gas molecules in the polymers, despite the fact that the POSS cages are chemically bound to the polymer chain in the necklace morphology. Amounts of the siloxane in the materials were the most significant factor for the gas permeation, suggesting that gas is transported exclusively through the DMS chain moieties. As a result, permeability of all gases in the ${\mathrm{DMS}}_{y}^{x}@\mathrm{POSS}$ membranes did not correlate significantly with kinetic diameter i.e., POSS molecules micro porosity influence was not distinctively observed. In general, POSS-PDMS nanocomposites perform similar to conventional organic polymers for gas separation (based on vicinity to upper bound). The trend observed with the increase of the DMS linker length provides an important understanding of the gradual property changes in the hybrid membranes. Applicability of these materials for small gases separation will also depend on the mechanical properties of the membranes at lower thicknesses (~100 nm) that are required to provide higher fluxes through the membranes [25]. We also believe these type of materials can be useful in other areas instead of PDMS (apart of the gas separation) e.g., microfluidics. Lower gas permeabilities would be beneficial there, especially for the systems where small molecular species can be absorbed in the material. As shown, in these DMS@POSS composites gas permeation can be controlled within a relatively broad range, by the composition.

## Figures and Tables

**Table 1:** Summary of the materials composition and the properties used to fabricate gas separation membranes.

Material Code	Average DMS Length, n	M_W_	M_N_	Crosslinking Ratio, MS-51:DMS@POSS	Siloxane Mass Content, %
${\mathrm{DMS}}_{15}^{4.4}@\mathrm{POSS}$	4.4	315,000	146,000	1:15	22.5
${\mathrm{DMS}}_{15}^{4.0}@\mathrm{POSS}$	4.0	110,000	81,500	1:15	20.9
${\mathrm{DMS}}_{5}^{3.4}@\mathrm{POSS}$	3.4	110,000	64,900	1:5	18.3
${\mathrm{DMS}}_{15}^{3.4}@\mathrm{POSS}$	3.4	110,000	64,900	1:15	18.3
${\mathrm{DMS}}_{30}^{3.4}@\mathrm{POSS}$	3.4	110,000	64,900	1:30	18.3
${\mathrm{DMS}}_{15}^{2.0}@\mathrm{POSS}$	2.0 *	76,700	40,100	1:15	11.7

* Fixed length of dimethyl siloxane.

**Table 2:** Glass transition temperatures of the ${\mathrm{DMS}}_{y}^{x}@\mathrm{POSS}$ composite membranes measured by thermal mechanical analysis (TMA) and dynamic scanning calorimetry (DSC).

Material	T_g_, °C by TMA	T_g_, °C by DSC
${\mathrm{DMS}}_{15}^{4.4}@\mathrm{POSS}$	~10	8.5
${\mathrm{DMS}}_{15}^{4.0}@\mathrm{POSS}$	18.6	16.5
${\mathrm{DMS}}_{5}^{3.4}@\mathrm{POSS}$	36.1	–
${\mathrm{DMS}}_{15}^{3.4}@\mathrm{POSS}$	36.1	41.0
${\mathrm{DMS}}_{30}^{3.4}@\mathrm{POSS}$	36.1	–
${\mathrm{DMS}}_{15}^{2.0}@\mathrm{POSS}$	~100	62.0

**Table 3:** Summary of the gas permeabilities in DMS@POSS membranes compared to Sylgard PDMS and commercial siloxane sheet.

Material	Average DMS Length, n	Permeability, Barrer				Siloxane Mass Content, %
		H_2_	CO_2_	O_2_	N_2_	
${\mathrm{DMS}}_{15}^{2.0}@\mathrm{POSS}$	2.0 *	87	121	16	4.8	11.7
${\mathrm{DMS}}_{5}^{3.4}@\mathrm{POSS}$	3.4	101	169	26	6.9	18.3
${\mathrm{DMS}}_{15}^{3.4}@\mathrm{POSS}$	3.4	99	201	27	7.6	18.3
${\mathrm{DMS}}_{30}^{3.4}@\mathrm{POSS}$	3.4	113	198	25	8.1	18.3
${\mathrm{DMS}}_{15}^{4.0}@\mathrm{POSS}$	4.0	143	374	50	15.3	20.9
${\mathrm{DMS}}_{15}^{4.4}@\mathrm{POSS}$	4.4	173	441	54	20.1	22.5
PDMS [22,18]	n/a	573	2850	533	250	n/a *
Siloxane rubber	n/a	1013	4343	828	387.2	n/a *

* Most likely close to 100% but the exact composition is unknown (commercial samples).

**Table 4:** Permeation parameters for nitrogen (N_2_) and carbon dioxide (CO_2_) of ${\mathrm{DMS}}_{y}^{3.4}@\mathrm{POSS}$ membranes with different crosslinking ratio derived from the Arrhenius plot in Figure 8. Data compared to PDMS reported in literature [22].

Material	P_0_, N_2_	E_P_ (KJ/MOL), N_2_	P_0_, CO_2_	E_P_ (KJ/MOL), CO_2_
${\mathrm{DMS}}_{5}^{3.4}@\mathrm{POSS}$	1.41 × 10^6^	30.3	1.19 × 10^5^	16.6
${\mathrm{DMS}}_{15}^{3.4}@\mathrm{POSS}$	1.17 × 10^6^	28.3	1.06 × 10^5^	14.8
${\mathrm{DMS}}_{30}^{3.4}@\mathrm{POSS}$	1.44 × 10^6^	30.2	1.95 × 10^5^	17.2
PDMS [18,22]	3.46 × 10^4^	12.2	4.81 × 10^3^	1.3
